# Strategic Customary Village Leadership in the Context of Marine Conservation and Development in Southeast Maluku, Indonesia

**DOI:** 10.1007/s10745-016-9829-6

**Published:** 2016-06-06

**Authors:** Dirk J. Steenbergen

**Affiliations:** Charles Darwin University, Darwin, Northern Territory Australia; North Australia Marine Research Alliance, Arafura Timor Research Facility, Darwin, Northern Territory Australia; Associate Fellow to the Asia Research Centre, Murdoch University, Perth, Western Australia Australia

**Keywords:** Customary leadership, Local governance, Community-based natural resource management, Marine conservation, Eastern Indonesia

## Abstract

This article critically examines engagements of village leaders in an NGO-facilitated participatory conservation program in eastern Indonesia. It explores how the program’s implementation strengthened leadership legitimacy of a dominant customary social group. Customary leaders ensured distribution according to particular norms, and in organizing village governance upheld specific interests and claims over natural resources. Villagers outside of the customary group remained marginalized in village governance, despite being important stakeholders. Findings reveal complex relationships between leaders and villagers that were strongly framed by orders of power and cultural history, which influenced how and to what extent peripheral groups participated. The case study concludes that village leaders can form effective avenues to deliver on conservation outcomes. However, in their preoccupation with maintaining leadership legitimacy, they may inadequately address dynamic intra-community tensions that could jeopardize long-term outcomes. Co-management partners can play significant roles in adapting management and prompting more inclusive governance processes.

## Introduction

Contemporary conservation and development practices increasingly recognize the importance of social justice and the rights of local/indigenous groups (Peterson *et al.*^2008^), and are progressively being implemented through more decentralized management frameworks (Bardhan 2002). In Indonesia the political decentralization processes introduced since 1999, with the enactment of the regional autonomy law,^1^ devolved formal administrative authority from central to provincial, district and subdistrict levels in part to catalyze more local involvement (Hadiz 2004; Resosudarmo 2004; Hidayat 2005; Yamazaki *et al.*^2015^). Local resource user groups in Indonesia are assuming significant roles in terms of both the physical implementation of conservation and natural resource management projects and the design of associated interventions and approaches (Hidayat and Antlov 2004; Fox *et al.*^2005^; Fritzen 2007). Although this is arguably positive, criticisms of community-based approaches often allude to substantial discrepancies between projected outputs from interventions and the reality of on-the-ground results (Cleaver 1999), and contribute to perceived ‘failure’ of community-based initiatives (Dasgupta and Beard 2007). Projects indeed are rarely implemented as stipulated in a-priori project plans; however this article is careful not to assume this as failure. Instead, it endeavors to show how local leadership strategies are applied to access, or appropriate, project resources toward community wide, dominant group and/or private interests in cases where external frameworks are flexible enough to allow village leadership to adapt them. It thus critically examines assumptions of self-interested leadership and ‘elite capture’ in natural resource management (Platteau and Gaspart 2003; Persha and Andersson 2014).

A primary objective of community-based conservation initiatives, as well as centrally coordinated rural development programs, is to achieve equity in benefit distribution and allow a fair allocation of resources so as to gain maximum breadth in beneficiaries and efficacy of interventions (Klain *et al.*^2014^). However, in establishing collaborative partnerships, often too little is understood of the local processes and politics of participation, or how roles of leadership are enacted, by whom and why. The influx of external resources into villages through such programs, in cases where different understandings and cultures of governance meet, may well produce contextually relevant hybrid practices (Cinner and Aswani 2007). As a result, ‘new’ governance and decision-making processes form that draw to varying degrees from official project-driven governing tools, administrative government, as well as customary law and norms (Cohen and Steenbergen 2015).^2^ Projects, and the local governance landscapes within which they are implemented, are actively transformed by local leaders’ roles in implementation, despite the defined objectives, methods and outputs stipulated in management plans (Morgan-Trimmer 2013). New collaborations develop through negotiations, amalgamations and consolidations at the interface between local and ‘outsiders’ interpretations of the roles and functions of leadership in a particular governance context. Critically examining how external resources are channeled through social groups in a community will reveal what structures and interests influence the direction of a project. This in turn can more accurately determine the value and sustainability of conservation project outcomes, as measured in terms of environmental change as well as their different impacts on various groups and individuals.

## Elite Capture, Leadership and Participation

Much of the attention in conservation and development program design is dedicated to creating systems that minimize free-rider behavior, corrupt practice or elite capture, and that maximize fair and targeted allocation or equitable benefit distribution (Bodin and Crona 2008).^3^ Although program designs often assume action by elite groups or individuals to be rationally driven by self-interest over collective goals (Gugerty and Kremer 2008), in fact these groups may employ strategies to maximize collective benefit in ways that adhere to local dominant culture and/or overcome contextual challenges (which may not have been accounted for in formal program design). In his review of approaches addressing elite capture, Wong (2010: 2) identifies ‘counter’ and ‘co-opt’ strategies to deal with elite capture. The former involve approaches that suggest a need to counteract elite tendencies through their exclusion from project management and design, based on assumptions that elites operate purely out of self-interest and thus are by definition impediments to collective good outcomes. The latter approaches suggest that cooperation with elite individuals or groups may provide opportunities to use existing leadership legitimacies constructively to incorporate otherwise disenfranchised and marginal groups. This aligns with more nuanced understandings of behavior by elites or leaders as being subject to complex relationships with, and accountabilities to, a wider society (Platteau and Abraham 2002). In the context of this study, local elites are identified as those customary leaders of a community, whose privileged positions are defined by family networks, land holdings, religious affiliation, personal history and personality (following Dasgupta and Beard 2007). These elites hold significant village-wide governing power and represent a majority group in an administrative village which also includes a minority group outside the customary (*adat*) network that despite its marginal position forms an important stakeholder group in conservation management contexts.

Studying impacts of collaborations across the interface between community leadership and external actors requires in-depth inquiry into the social and political complexities within a village leadership constellation, and between leaders and members of other social groups. This in turn reflects how and why particular individuals, as ‘elites’ or otherwise, appear as community leaders. Relations between different leadership constellations that flow from underlying social divisions, by no means suggest that the social groups identified here are homogenous and without internal contestation. In focusing on current dominant leadership constellations of the village’s customary (*adat*) core families the study highlights potentially contentious problems of representation at village level (Baland and Platteau 1997, 1998; Lund and Saito-Jensen 2013) so as to understand how different groups operate under current leadership conditions. To make sense of village leadership, the study acknowledges that leadership materializes from more than simply the actions of individuals in leadership positions; aligning with Case *et al.*’s (2015: 3) understanding of leadership that argues for more “complex, rounded, and nuanced interpretations of leadership practices, which are sensitive to cultural contexts, plural perspectives, and contestation”. The dynamic nature of these constellations, and the changing relationships which predispose various alliances or oppositions, thus warrants recognition of the temporal and social contextual limits of the findings from this case study.

**Fig. 1:**
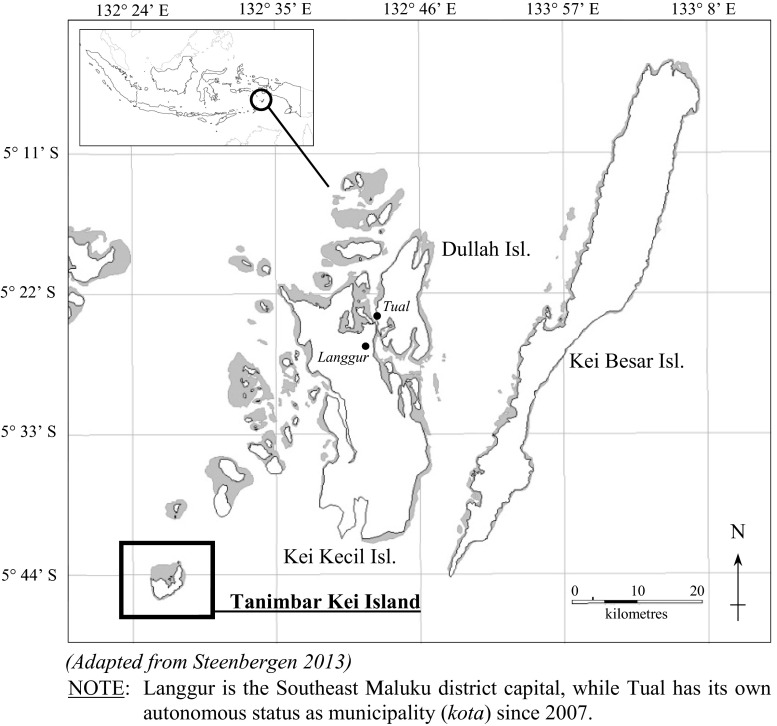
Map of the Kei Archipelago (Southeast Maluku district in Maluku province)

The study draws from ethnographic research carried out in the remote island community of Tanimbar Kei, located among the Kei islands in eastern Indonesia (Fig. 1). Like many remote communities in Indonesia, leaders here need to address potentially dichotomous objectives – in particular to advance conservation and development without pursuing these at the cost of livelihoods, resource sustainability or loss of important customary practices and values. It is in examining which practices and norms are given precedence over others that group-specific sets of interests become important. The dilemma of negotiating what is beneficial, acceptable or intrusive change is highly contextual and dependent strongly on who in the community is asking the question, and with what short and long term implications. The analysis therefore seeks to show to what extent, and in what ways, local leadership strategies reflect a need to legitimize leaders’ positions to a wider local constituency. It also needs to account for the fact that ‘legitimacy’ may reflect the perspectives and interests of a dominant group, in this case the customary (*adat*) group in Tanimbar Kei, more than that of the village as a whole. This leads to the question of whether a dominant group’s authority offers an appropriate starting point for conservation projects (Labonte 2012; Steenbergen and Visser 2016), and whether such alignments can be conducive to the sort of institutional bricolage that Cleaver (2001, 2012) suggests effective adaptive strategies could be built upon.

The conservation program at the center of this study is a community-based marine conservation program involved in establishing a Locally Managed Marine Area (LMMA) in the village of Tanimbar Kei. This program involves an Indonesian NGO known as the Indonesian Locally Managed Marine Area Network (I-LMMA).^4^ I-LMMA follows implementation of local marine conservation programs along community-based adaptive management principles (Govan *et al.*^2008^ ) that are receptive to the particular local conditions of communities which have tended to rely on longstanding customary institutions (I-LMMA 2011). The program is particularly relevant, since its organizational structure and approach to collaborative conservation offers partners (i.e. coastal communities) significant autonomy, implying that community actors have a primary say in how projects are managed and implemented. This provides the opportunity to examine if, how and why local adjustments to management of a program occur.

## Methods

The study is based on data collected in the community of Tanimbar Kei, during a seven-month residence there between August 2010 and February 2011, followed by five subsequent visits of up to 3 weeks each between 2013 and 2015. My ethnographic engagement with villagers in daily livelihood activities, through participant observations and frequent conversations with key informants provided most of the data used here. Semi-structured interviews were carried out with villagers (*n* = 55) during the initial data collection period to learn about people’s perception of, and participation in the village LMMA program. In this same period a survey on local leadership and village governance was carried out with 42 households in the village, which were randomly selected after stratifying the data population according to hamlet size. Although the name of the village is used here, the identities of my respondents remain anonymous, and only public positions are named when referring to individual leaders.

## Tanimbar Kei

In a country made up of over 18,100 islands, with an estimated 60 % of its 250 million people living on small islands or within 50 km of any coastline (CTI-CFF 2009), dependence on coastal resources in Indonesia is high. The archipelago’s political center revolves strongly around the island of Java, with the remote eastern-most regions of Maluku and Papua being the least populated (Cribb and Ford 2009). However, the exceptionally rich coastal waters in this eastern frontier not only sustain the nutritional, livelihood and cultural needs of millions of island people, but over various periods have attracted diverse international and domestic commercial fishing fleets (Novaczek *et al.*^2001^). As a result the governance seascape in contemporary Maluku sees coastal resources subject to various governing systems including central government policy and local customary law (Thorburn 2000).

Tanimbar Kei Island is located some 4 hours by motorized fishing boat (*body*) from the Kei Archipelago’s main island of Kei Kecil (Fig. 1), and falls under the Kei Kecil Barat Subdistrict as part of the Southeast Maluku District in Maluku Province. The island’s village shares its name and is made up of approximately 125 households with a population of about 507 people (Kecamatan Kei Kecil Barat 2010). The village is administered under a single village (*desa*) administration with no administrative hamlet (*dusun*) subdivisions, despite there being significant historically and physically distinct groupings within the village.^5^ The main settlement for example is divided into a traditional older section on higher ground and a newer section along the village’s foreshore. Another smaller settlement, known as Mun, of about 16 households is located some three kilometers north east along the northern coast and is made up largely of migrant settlers of Bugis background. Although Tanimbar Kei identifies itself as a Hindu village, four religions are represented; including in order of size: Hindu, Protestant and Catholic in the main settlement, and Muslim in the Mun settlement.

**Fig. 2:**
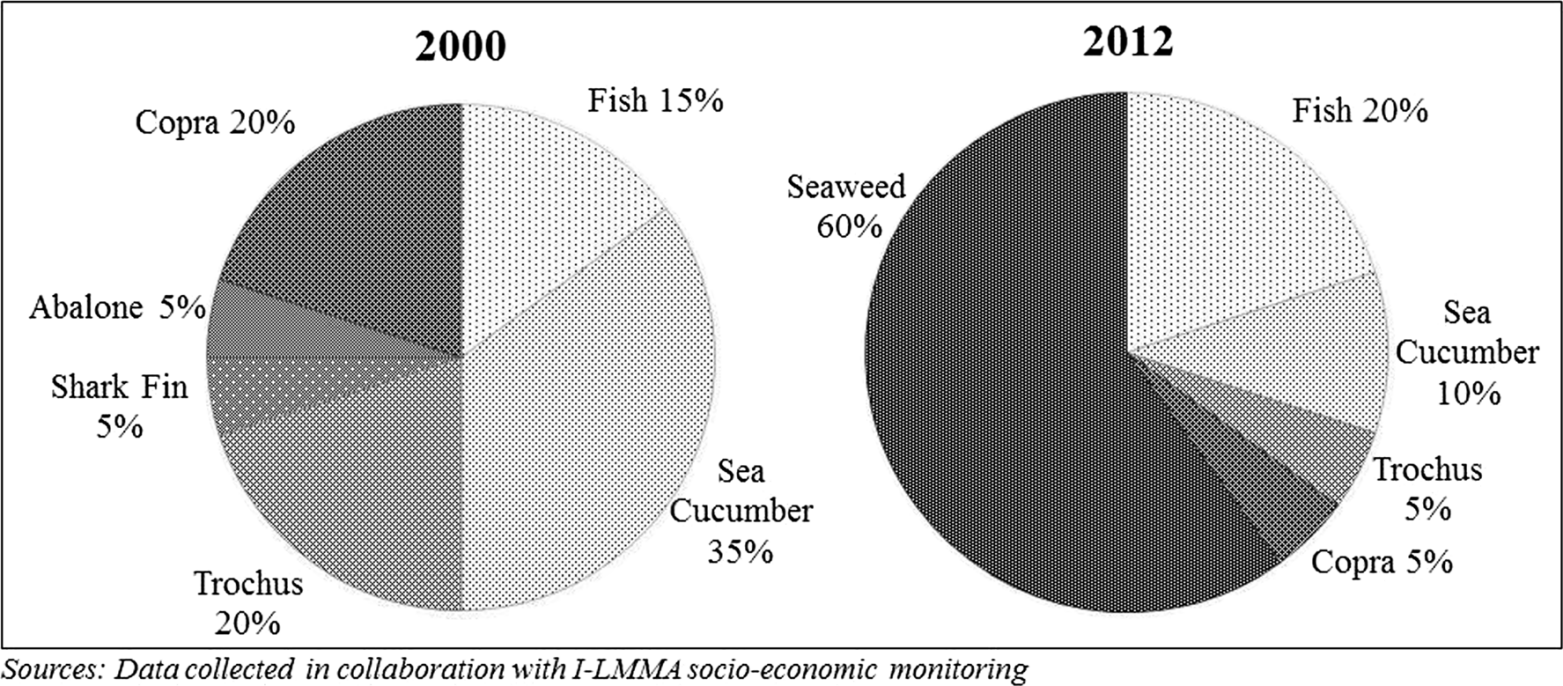
Average natural resource-based income distribution of Tanimbar Kei households in 2000 and 2012

Livelihoods on the island largely depend on the surrounding marine environment. As indicated in Fig. 2, since 2005 the local village economy has shifted focus toward the cultivation, processing and trade in seaweed (carrageenophyte seaweed of genera *Euchema*, referred to locally as *agar agar*), to become the main source of household income across the village. Many households also still tend coconut plantations to produce and sell copra to middlemen, albeit of lesser importance than before 2005. Fish and other reef-based marine resources are an important part of people’s staple nutritional intake. Given the distance to the larger Kei Islands and lack of cool storage technology, only a small portion of total village catch is traded and only by a few households selling to other households in the village. Aside from marine oriented livelihood activities, households typically maintain small agricultural plots where they plant limited hardy root crops (e.g., cassava), fruit (e.g., papaya) and vegetables (e.g., string beans). The island’s low-lying limestone substratum means soils are infertile and opportunity for extensive agriculture is limited. Basic food staples like rice are bought from markets on Kei Kecil.

Both men and women take part in most land-based economic activities (i.e., copra or other food production), albeit with different responsibilities. Marine-based activities beyond the mudflats are exclusively carried out by men (e.g., reef and pelagic fishing), while women share in activities within the intertidal zone (i.e., inside the confines of the fringing reef) such as seaweed farming and gleaning for mollusks. Other more specialized income sources on the island include boatbuilding, community shops (*kios*), and bulk transport to Kei Kecil. There are a handful of villagers that work as civil servants, but only one resides permanently in the village (other civil servants in the village include teachers and a health worker posted from elsewhere).

In comparison to mainland villages Tanimbar Kei lacks physical infrastructure. With no connection to an electricity-grid households must produce their own power from generators. The village offers up to primary school education and a health center with a fulltime health worker who can provide basic treatment and maternity care. For secondary school education or more specialized healthcare villagers must travel to the district capital Langgur on mainland Kei Kecil. Fresh water is exclusively sourced from ground wells, whereby a single well located near the Muslim settlement of Mun provides the island’s main drinking water supply since most ground wells are brackish and used for washing. Mobile phone reception reached Tanimbar Kei only since late 2012, which has allowed for more frequent communication with the mainland.

Considering the small island’s remoteness and people’s dependence on the marine environment, livelihoods on Tanimbar Kei are highly vulnerable.^6^ As similarly reported in other cases of remote and vulnerable societies (Bankoff 2007; Beekman *et al.*^2009^; Schwarz *et al.*^2011^), the different groups of households that make up Tanimbar Kei village exhibit exceptionally high internal social cohesion. This reflects in the persisting recognition amongst Tanimbar Kei’s residents that they all are subject, in varying degrees depending on their social position, to the strong customary system (*adat*) described in Barraud’s (1979) extensive anthropological study of Tanimbar Kei. This is despite the withering away of much of the traditional governance systems in neighboring island communities closer to Kei Kecil. Traditional governance processes on Tanimbar Kei continue to revolve around customary social organization (Barraud 1979: 87). Twenty-three patrilineal social units referred to as ‘houses’ or *rahan* (Barraud 1979: 87–94) form the entirety of Tanimbar Kei *adat* society. Each of the 23 *rahan* belong to one of three superior units known as *la-owan*, which are led by what are believed to be Tanimbar Kei’s three founder lineages. Together, the 23 ‘houses’ form the *adat* core of the village. The authority of a particular *rahan* over another *rahan* within a *la-owan* is context dependent, since responsibility over particular matters is customarily assigned to certain *rahan*. Although over the last decades the village has expanded to include households of migrants or extended family units, members of the 23 houses or *rahan* still make up the majority of the village. About 75 % of villagers claim some form of close association through direct kin ties or marriage, making them eligible for particular inheritances associated with a *rahan*, including rights to land and *adat* responsibilities.

Although accounts from villagers showed that *adat* leadership, prior to any conservation program, indeed influenced how people used resources, this was not particularly motivated by conservation. The opening and closure of marine resource harvests in times of need, for example, was determined by *adat* leaders who communicated with spirits to ask permission or attain their blessing (Cohen and Steenbergen 2015). However, a notable intervention by *adat* leaders occurred following extensive degradation to the island’s reefs and fish stock in 1990s due to bomb fishing by outside fishers and particularly due to impacts of a live fish trade company that operated over their reefs. Under an agreement with local *adat* leaders the company employed Tanimbar Kei fishers to collect live fish, and paid an annual concession fee to the *adat* leaders. The destructive effects on coral of the potassium cyanide supplied to the divers by the company showed after several years, in addition to particular reef species (e.g., Napoleon wrasse - *Cheilinus undulatus*), being all but fished out by 1998. In that year *adat* leaders reportedly terminated the collaboration and collectively demanded that the company leave. In the years following their exit, little consolidated effort materialized amongst *adat* leaders to improve marine areas due to what a local leader described as “a lack of technical knowledge in the village” on how to address the acute ecological degradation. Their lack of capacity to address the degradation eventually led to I-LMMA’s invitation by the village head in 2005.

## Local Centers of Governance in Tanimbar Kei

The seven principles of *Hukum Larvur Ngabal* (‘laws of the red blood and lance’)^7^ persist as the foundational structure of customary law and order on the Kei Islands (Laksono 2000; Adhuri 2006, 2013), particularly in recent years after recognition of *adat* institutions under Law No. 32/2004 on regional government (Thorburn 2008). The seven principles set out values associated with individual moral conduct, mutual respect, property ownership and the strongly hierarchical system of social ranks. Although Tanimbar Kei, like all Kei communities, is subject to these broader laws, Tanimbar Kei society developed a distinctly different niche set of principles (Barraud 1979). Customary law here is dictated by perceived connections to the spirit world and materialized through villagers’ relations with one another and their marine environment.

The customary governance structure around decision making in contemporary Tanimbar Kei is still rooted in the *rahan* constellation and enacted through a traditional council made up of heads of each *rahan* who are appointed based on the patrilineal inheritance. The involvement of all *rahan* heads suggests there is more consensus-based decision-making here (at least within the *adat* core group) compared to elsewhere on the Kei islands. Not all village matters are necessarily brought to this council, however. Smaller intra-village disputes are for example addressed by particular leadership figures associated with the group where the dispute occurred, as is the case with Mun, where the Muslim leader assumes a primary role in conflict resolution. Religious leaders from all four religious groups play a significant role in local social conflict resolution, although at a village level they function below the authority of customary *adat* leaders. More recently legal matters and inter-village conflicts have increasingly become the domain of the official village administration (*desa dinas*), but as demonstrated in the following section there is strong influence and representation of *adat* in the village administration.

The establishment of Suharto’s New Order regime in 1966 saw the implementation of an Indonesian central state bureaucratic system that extended down to village level, consolidated under Law No. 5/1979 on village government. In Tanimbar Kei village, governing structures were instituted whereby local *adat* systems were superseded by the national Indonesian legal system. In part enabled by its remote location, Tanimbar Kei leaders remained *strongly* linked to their traditional governing institutions and maintained these substantively intact. For example under Tanimbar Kei’s customary *adat* structure (before central state influence), the role of village head could only be occupied by individuals from one *rahan* lineage and was historically inherited along patrilineal lines, rather than through open elective processes. It furthermore represented more of a diplomatic responsibility as the ‘face of the village’ in its engagement with outsiders, and was thus not regarded as a singular point of authority, nor did it involve administrative responsibilities or presuppose extra benefits like a stipend. This contrasts with official village administration under Indonesian law, which is locally understood as a body of externally derived authority backed by central government.

Tracing back bureaucratic village leadership in Tanimbar Kei since its implementation in the late 1970s shows that these positions have always been occupied by men from the 23 *rahan* of the *adat* core. The first appointment of an official (*dinas*) village head was reportedly around 1975, and saw the head of the *rahan* associated with the customary role of village representation in external engagements assume this position. Since then the position has been filled by others from the *adat* core structure following more elective processes, although eligibility for candidature remained implicitly dependent on inherited entitlements under *adat*, and more recently on being Hindu. In one instance the son of a previous village head made claim to the position of village head, however his challenge was rejected by village elders since he had converted from Hinduism to Christianity (Protestant), and thus was no longer eligible to lead Tanimbar Kei. Similarly, the official village council (*Badan Permusyawaratan Desa*, BPD), as the main village planning and consultative structure under Indonesian law, was at the time of the research dominated five-to-one by elders or representatives from the 23 *rahan adat* core. As a result customary village leadership norms still strongly shape village governance and steer engagements with external institutions and organizations.

The administrative parameters associated with official village government (e.g. fixed terms, democratic election procedures and embeddedness in national bureaucratic frameworks), have in more recent decades bureaucratized the village head position. Nonetheless, among the local governing centers in Tanimbar Kei, the customary *adat* core social order, and the powerful *rahan* heads that collectively comprise its leadership ‘elite’, maintain the highest level of leadership legitimacy. The hierarchy among *rahan* heads is determined in part by the specific roles historically bestowed on their *rahan*, in particular the three leading *rahan* of the respective *la-owan*, but appears also to be influenced by personality. One of the heads of the three leading *rahan*, for example, possesses significant authority through his inherited role as ceremonial master of important *adat* rituals involved in annual collective millet cultivation. His authority over *adat* matters in particular is very strong and is embedded not only in his authoritative presence, but also in the respect among villagers of his exclusive knowledge of, and connections to, the spirit world. Another of the three leading *rahan* heads, claims lesser authority over *adat*-ritual matters, but is a prominent leading voice in village decision-making, planning and development. He too assumes significant respect from villagers, largely based on his personality and proven leadership track-record over the years.

**Fig. 3:**
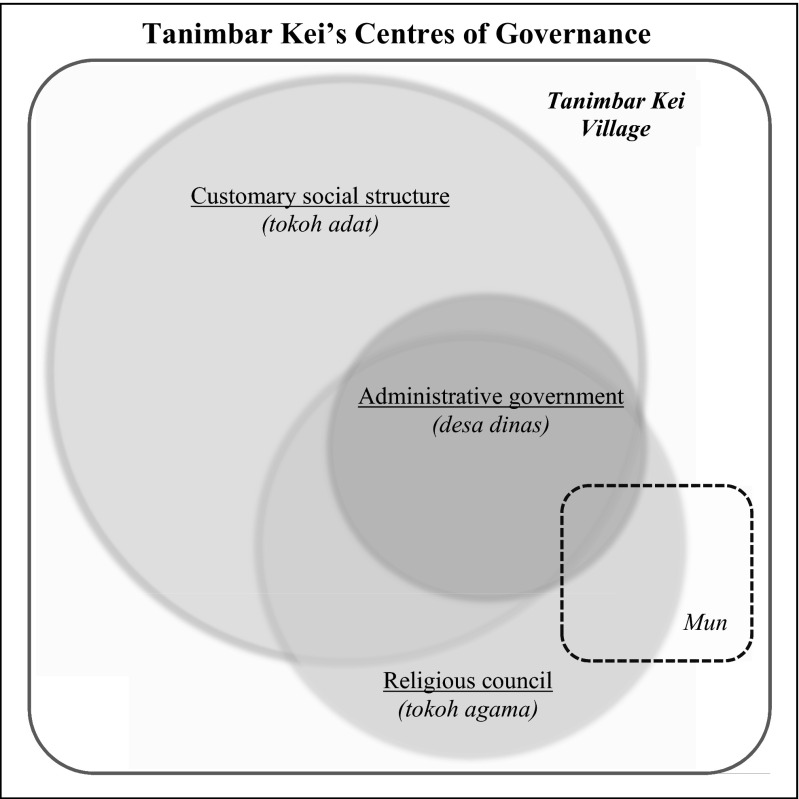
Schematic representation of Tanimbar Kei’s governance centres based on village leaders’ association to one or more of the village leadership institutions, with indication of Mun’s relative representation in village-wide governance bodies

The three leadership bodies often associated with official village governance in Indonesian villages, namely the village government (*desa dinas*), the religious council (*tokoh agama*) and the traditional council (*tokoh adat*), do not stand on equal ground in Tanimbar Kei. As schematically presented in Fig. 3, *adat* leadership in Tanimbar Kei is dominant and strongly embedded in *adat* identity amongst the 23-*rahan adat* core group. Primary membership in this group is defined by direct kinship connection and religious affiliation to Hinduism. Where there are examples of *adat* kin members that have converted to other religions, these individuals may still identify with the *adat* community, take part in *adat* ceremonies and make claim to particular land, but they cannot become head of a house or fulfil *adat* leadership roles. Albeit secondary to *adat* leadership, religious leaders from all four religions on the island are important figures also and have come to be integrated in one way or another into village governance. For example, the final institutionalizing of various village regulations requires sign-off by the religious council, next to the traditional council and village government. Under I-LMMA’s facilitation the village conservation program was first to apply these requirements, however since then these have been independently applied to legitimize agreements made in village-wide government development initiatives.

The administrative village government (*desa dinas*) is a more recent institution, yet increasingly gains currency as an avenue to access resources and infrastructure through national development programs. The vast majority of its staff belongs to the *adat* core group. However several well-respected figures, as for example the Catholic Church leader, are also involved. Originally from a village on the mainland of Kei Kecil, he married into an *adat* family and has ever since lived in the *adat* core section of Tanimbar Kei. Being well educated and from a high ranking social order on Kei Kecil^8^ has likely aided his integration. However, many claimed his authoritative, calm personality and his reputation for fair judgement gave him his prominent position in village government and in the LMMA village program.

Integration of the smaller Muslim hamlet of Mun in village-level leadership does not extend beyond involvement in village government of the Muslim leader (*imam*) and several elders (who in fact make up part of the customary *adat* group and are still recognized as important figures, but have settled in Mun through marriage). Without administrative hamlet status there is no separate body representing interests for Mun residents specifically. As a result most involvement in village government matters by these elders is primarily through the village administration’s consultative meetings rather than by an integrated process that recognizes a formal role for Mun in decision-making. This is evident also in their low participation in the village LMMA program.

## Indonesian Locally Managed Marine Area Network

The village LMMA conservation program functions under a co-management partnership with the Indonesian Locally Managed Marine Area Network (I-LMMA). Being a relatively small NGO, I-LMMA regards its assets as largely embedded in the community programs it mentors. Although focus remains small scale, I-LMMA’s larger vision is “to create and share a community-based, sustainable, and fair marine resource management model for coastal areas and small islands in Eastern Indonesia” (I-LMMA 2011: 1). Tanimbar Kei’s LMMA program aims to assist in developing sustainable local management regimes for marine resources primarily for the socio-economic benefit of the community as a whole. The first collaboration started in 2005 when Tanimbar Kei’s village head participated in a workshop on sustainable sea-cucumber cultivation facilitated by I-LMMA. Shortly afterward he invited I-LMMA to the island to discuss ways to address the state of their marine environment following the ecological degradation and overfishing that occurred with the live fish trade company that had operated in Tanimbar Kei. This eventually led to the inception of the village LMMA program a year later wherein focus was strongly on rehabilitating damaged reefs. Since then targets have been developed to incorporate more sophisticated management regimes over marine resource stocks to enhance sustainability and economic viability of local people’s marine-oriented livelihoods.

**Fig. 4:**
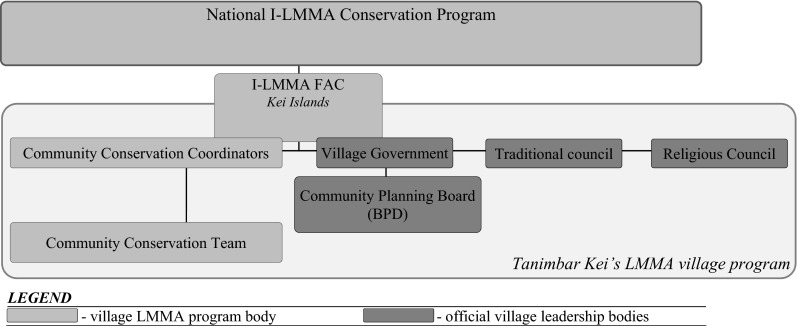
Official organizational structure of the co-management partnership between I-LMMA and Tanimbar Kei

The national I-LMMA program is coordinated by a small team of four fulltime employees. Additionally four focal area coordinators (FAC) are employed to oversee and coordinate program activities in each of I-LMMA’s focal areas.^9^ Beyond these positions there are no fulltime paid positions. At community level all programs share a common organizational configuration (Fig. 4): three locally-chosen conservation coordinators take a lead in facilitating and managing in-village activities, and a conservation team of community volunteers carries out program activities (e.g., conservation interventions, monitoring). According to I-LMMA protocol, the community conservation coordinators are elected by the community where the program is implemented and receive a small monthly honorarium for their coordinating duties. Members of the community conservation team are volunteers who may receive secondary benefits like meals during activities or occasional remuneration at a daily rate (e.g., for monitoring and mapping activities). The current FAC for the Kei Islands is from Tanimbar Kei and doubles in his role as one of the three community conservation coordinators. Together with the two other community conservation coordinators they form the main connection between Tanimbar Kei and the I-LMMA national program.

## LMMA Village Program Outcomes

The program has designated a 40,000 ha (400 km^2^) marine zone around the island as an LMMA, with one small four hectare permanently closed no-take area designated as a nursery for trochus shell (*Trochus niloticus*) population regeneration. Annual trochus shell harvest outside of this no-take area moreover has been reduced from the previous six-month opening to a five-day opening period. Annual population monitoring activities carried out in the first 4 years after the implementation of the harvest restriction in 2006 showed a significant annual increase in average trochus size (from 7 cm in 2006 to 13 cm in 2010), and a consistent rise in the total annual trochus harvest (from 814 kg in 2006 to 2334 kg in 2010). Similarly, 5-yearly coral monitoring activities that mapped coral cover around the island between in 2006 and 2011, indicated significant growth of hard corals in areas that had previously been damaged.

These ecological and socio-economic monitoring activities are carried out by the community conservation team. Besides having to report on management effectiveness, these activities are meant to develop local skills in identifying and understanding drivers of local change around the island and build local capacity for project and database management. These activities are stipulated in annual work plans that are formulated and managed by the in-village LMMA team with I-LMMA’s support.

The total LMMA area corresponds with the traditionally owned marine territory of the Tanimbar Kei people, all of which falls within the four nautical miles under official district jurisdiction. The community has gained legal recognition for the ownership and management rights over the area from subdistrict government, through facilitation provided by I-LMMA. In a similar vein, the program has facilitated the formulation of a set of official resource use regulations (*Peraturan Desa Tanimbar Kei*, No 1, 2008). This mandates Tanimbar Kei, through village government, to sanction anyone in violation of the regulations. Using these, the community manages outsider fishers who harvest marine resources without permission from *adat* leaders or who use destructive fishing techniques defined as illegal in state regulations, which are reiterated in the village regulations.

More recent regular seasonal visitation by outside fishers, most commonly Bugis and Butonese fishers, has prompted the formulation of a small scale fisheries (SSF) management plan. Since 2013 the LMMA has become subject to a rotating system along four equal quadrants that dictates fishing access annually, so that in any given year one quarter of the LMMA is closed for fishing. Implementation of this rotational closure has been agreed upon but has yet to be enforced.

Since 2012 the village LMMA program has implemented a concession system, whereby seasonal fishing ‘licenses’ are sold to outside fishers. This has created a significant source of collective income, which is managed by the coordinators in conjunction with *adat* leaders. At a meeting involving *adat* leaders, I-LMMA staff and the LMMA village program team members (which therefore also included village government staff), it was agreed that the income would firstly contribute to financing ongoing activities of the LMMA village program and secondly be set aside as collective savings. These savings could be drawn upon in several instances, but only with consent of *adat* leaders and the LMMA village program team. It was agreed at the meeting that the funds could be used to support communal events or activities like the annual new year festivities (commonly celebrated village-wide across Kei); to support poor households in crisis (i.e., acute food insecurity or family loss); and to support students who are in their final year of tertiary education (as the high expenses in the last year was a main reason students in the past did not complete their studies). Conditions were moreover established that only residents of Tanimbar Kei were eligible to appeal for these funds (in the case of tertiary education support, students’ parents had to be living on the island). In the first year, these funds were primarily channeled back into LMMA program activities to cover operational costs. However in the second year, next to covering operational program costs, the funds supported one tertiary student in his final year, helped another family overcome a family crisis and provided households village-wide with rice after government food relief provisions did not eventuate. Although rice provisions were handed out across the whole village, including Mun, the two cases involving family-specific support both involved families from the 23 *rahan* structure. Other ‘non-*adat*’ families were technically eligible for such support and several were arguably in need of it, however none of these families applied for such support nor were they put forward by a third party.

## Tanimbar Kei’s Leadership Strategies through Partnership with I-LMMA

Tanimbar Kei’s collaboration with I-LMMA developed from an initial series of meetings between I-LMMA staff and the administrative village government staff. In commencing the collaboration with Tanimbar Kei, I-LMMA emphasized the program’s objective to establish marine resource management regimes through customary governance structures instead of through the administrative village government, which was the rule in state-funded development programs. The village LMMA program would need to be embedded among village level institutions and coordinated onsite rather than by I-LMMA from its offsite headquarters. Customary leaders in Tanimbar Kei were particularly supportive of establishing the coordinating base in the village since past NGO development projects, initiated through village government structures, had repeatedly led to disappointing outcomes and left villagers disheartened. Villagers described how on several occasions external NGO staff collaborated with the village government at the time to carry out rural appraisals in the community. The appraisals would inform development proposals for funders, which the NGO promised to implement once approved. However, these would rarely come to fruition even though many respondents suggested that the NGO in fact often received money to do so. The process remained vague for most villagers as participation was focused around village government staff and rarely developed further than appraisal stages. As one elder noted:

We [Tanimbar Kei people] had several NGOs come to us to start projects […], we always agreed, but they [local NGOs] always only worked closely with members of the [administrative] village government because they thought those were the most important people in the village. That is not so in Tanimbar Kei.

(Interview with *rahan* elder, Tanimbar Kei, November 2010).

These NGO programs regularly bypassed customary leaders who hold most authority in the village. Many villagers, including village government staff at the time, regarded decision-making in programs not involving *adat* leaders as tedious since they often needed to be consulted separately before decisions could be made, resulting in indirect and hesitant leadership over project implementation.

At communal meetings that were initiated by senior members of the *adat* community to commence the LMMA program, three villagers were elected into community conservation coordinating positions. These meetings, although open to all villagers, were largely attended by villagers related through the 23 *adat* houses, and from Mun only the Imam was present. The three individuals were selected by a naturally *adat*-dominated audience from a shortlist of ten villagers who had been identified earlier by prominent *adat* leaders together with village government staff (most prominently the village head) on the basis that they possessed necessary authority and leadership qualities. With exception of one individual (the Catholic community leader), all individuals on this list were bound through kin ties to the *adat* core. The selection procedure for the community conservation team that was also formed during this meeting involved more specific selection criteria specified by I-LMMA. As a requirement from I-LMMA to ensure ‘broad capture’, the community conservation team had to be made up of seven core youth members from all parts of the village, while maintaining an open structure to allow free participation in activities by all (*adat* and non-*adat*) villagers. I-LMMA furthermore insisted on the inclusion of two women and a representative from Mun as core members. However those present at the meeting appeared to have steered the selection process towards an *adat* preference, since all chosen core members of the community conservation team, including the two women, were part of Tanimbar Kei’s *adat* core kin structure (although some had different religious backgrounds). The selected representative of Mun was also from an *adat rahan*; having married a woman from Java, he had chosen to settle in the Muslim community of Mun at the wish of his wife. Local leaders justified it on the grounds that this individual came to the main village frequently to see his family as opposed to other Mun inhabitants, thus allowing him to participate in meetings when called. Given that only the Imam attended the meeting, Mun representation in the community conservation team following this meeting was predictably low. While their absence at the meeting was interpreted by many *adat* leaders as disinterest, the Imam attributed this to reluctance among many Mun residents to confront *adat* leadership.

I-LMMA’s focus on developing local autonomy and leadership capacity formed its main strategy to gain community interest, an approach that appealed to many in Tanimbar Kei. Support for this approach was unsurprising, considering that villagers frequently made reference to their collective identity as being distinct from the wider Kei culture. In that vein, I-LMMA sought to develop in-village social and environmental project assets through proven collaboration and commitment of enthusiastic villagers, rather than initiating intervention with an injection of capital and physical assets. Given that the project initiation stages ran primarily through *adat* leadership, responsibility for decision-making in the program remained strongly aligned with the perspectives and interests of customary leaders. This gave the program a strong local *adat* identity. The program secured further support once the first formal recognition by subdistrict government of tenure rights over the marine territory was realized, allowing Tanimbar Kei leadership to take tangible measures to control outsider access.

The village LMMA program was regarded by many respondents to have helped elevate living standards. It is worth noting that the village LMMA program’s inception coincided with the local seaweed cultivation boom after 2005 (Fig. 2). It led to an exponential growth in the island’s local economy, whereby living standards improved and villagers no longer relied on temporary wage labor from outside the village, meaning most family members returned permanently to the village. The economic boom also meant livelihood focus for most households shifted away from coral reefs, which meant that restrictions on marine resource harvesting instituted by the program did not meet the same resistance as experienced elsewhere. More specifically though, many leaders noted that the program had reinforced local traditional leadership structures in what was seen as a critical time of increasing globalizing influences through increased cash economy, new technologies, youth pop culture and media reaching Tanimbar Kei. The expansion of the village LMMA program built on local support through its alliance with dominant *adat* leaders from the 23 *rahan*; rather than through other potential links which might have included Mun’s fishing-oriented minority Muslim community.^10^ Some *adat* leaders referred to the program as their own local initiative, downplaying I-LMMA’s facilitating support, and the program was frequently used in narratives of the communities’ distinctiveness from neighboring communities,

We [Tanimbar Kei people] are not like them [villages on neighboring islands] […] you can see we are the only village that have maps and fishing rules which we made ourselves […] we take care of ourselves.

(Interview with *rahan* elder, Tanimbar Kei, November 2010)

In contrast members of Mun households often spoke of their lack of knowledge of the program when asked to comment on particular interventions, “We [Mun residents] are not the correct people to ask about these things [implementing trochus management interventions][…] we are not really involved in what they [villagers from the main village] do” (Interview with Mun resident, Tanimbar Kei, February 2011). Mun households did not engage in trochus shell collection or trade as part of their livelihood strategy, therefore restrictions on trochus shell harvest triggered neither interest nor resistance among Mun residents. When asked, Mun households did however express concern regarding the rotational fishing closure system, for two main reasons. First and foremost the access restriction to regular fishing grounds they visited would possibly mean a loss in income, and secondly these restrictions meant that they could no longer offer fishing access to visiting Bugis fishers. These reservations, however did not lead to collective efforts by Mun residents to raise this issue with village leadership or with the community conservation team, likely because the rotational closure system was yet to be enforced implying that their fishing had yet to be impinged upon.

## ‘Winners’ and ‘Losers’ from Conservation Program Outcomes

In understanding the *adat* leaders’ dominant role in the LMMA village program, it is important to take note of the program’s impacts in terms of conservation outcomes and benefit distribution (Table 1). Examining which groups have benefited from activities, and which have been negatively affected or are likely to be affected in the long term, indicates not only the appropriation and mediation capacities of the *adat*-dominated leadership but also the position of the minority group and what implications that might have for future program management.

**Table 1:** Distribution of benefits and responsibilities associated with Tanimbar Kei’s LMMA village conservation program

Main conservation outcomes	Immediate ‘winners’	Immediate ‘losers’	Unaffected (immediately)	Affected in long-term under status quo conditions
Trochus harvest management	Trochus shell harvesters (*adat families*)	None (*recent shift to seaweed farming meant little focus on trochus*)	Mun	
	Increased total annual harvest	Restrictions accepted	No interest or dependence on trochus	
Honorary employment & skills/knowledge improvement in natural resource management	LMMA community conservation team (*all members from adat families*)	None	Mun	Mun (*minimal interaction with LMMA team means little formal or informal information exchange*)
	Monetary benefits & skills	Open invitation for participation in community conservation team	Not involved and not particularly interested to get involved	With no participation, less skill development to foster potential future engagement
Legal ownership & legal tools to address illegal fishing	Whole village (*consensus against destructive fishing*)	Outside illegal fishers (*thus far no prosecution of local residents*)		Mun
	Secured access for all residents & decreased illegal fishing (less damage & improved marine environment)	Enforcement of village regulations restricted to outside fishers		With strong & extensive diaspora links through outside Bugis networks, apprehension of outside Bugis fishers could spur resistance among Mun residents
Collective income from fishing concessions & sanctioning	2 *adat* families (*education & poverty support*)	Outside seasonal fishers		Mun
	Whole village (*rice provisions & new year celebrations*)	Bugis & Butonese flying fish roe harvesters^a^		With strong & extensive diaspora links through Bugis networks, imposition of levies on outside Bugis fishers has already incurred resistance among Mun residents

^a^In contrast to the previous free open access arrangement, under the new SSF management outside harvesters pay for seasonal access

With the exception of outside Bugis and Butonese fishers, Table 1 presents no clear immediate ‘losers’ related to conservation interventions. Four factors play a role in accounting for this. Firstly, restrictive measures associated with trochus harvest, where one would expect resistance, were readily accepted throughout the village largely due to the prominent livelihood shift towards seaweed farming which relieved local peoples’ dependence on marine resources. Secondly, minority groups like the Mun residents, who did not necessarily share *adat* interests, nevertheless were less likely to resist restrictions since that might jeopardize arrangements with the *adat* leadership which thus far allowed for continuity of their livelihood pursuits. Thirdly, the cases where collective benefits from concessions were distributed across the whole village (i.e. rice provision and the new year celebrations) indicate that customary leaders did acknowledge the needs of other groups. So although arguably limited, these other groups did receive some form of benefit. Lastly, in the context of *adat-*dominated leadership, I-LMMA’s criteria for participation of peripheral groups, although steered by *adat* leaders, did urge those leaders to incorporate measures for wider participation.

In examining who ‘won’, the *adat* core group certainly benefited significantly from the LMMA village program. With legal rights and village regulations crafted around *adat* norms and *adat* members taking up program positions (implying higher exposure to opportunity for skills development), suggests an active appropriation of opportunities by *adat* leaders. This sway in the balance of authority and potential benefit distribution in favor of the *adat* core group, and the consequent points of tensions with minority groups that may emerge, suggests minority groups may increasingly find themselves disadvantaged. Indication of this tension already appeared during the community conservation team’s catch monitoring activities, which involved gathering catch landing data from outsider seasonal fishing groups. Many Mun residents have Bugis ancestry, which means that they maintain significant ties across extensive Bugis diaspora networks. In their seasonal fishing activities around Tanimbar Kei outside Bugis fishers use Mun as a base of operation. Whereas outside Butonese fishers were seen by the community conservation team to cooperate willingly, significant antagonism existed towards Bugis fishers. LMMA conservation team members noted that cooperation with Bugis fishers was difficult and complicated by their links to Mun.

Getting information from them [Bugis fishers] is always more difficult [than from the Butonese fishers], they do not like to work with us and are unreliable […], they always stay in Mun for a long time especially during the flying fish season, but they do not want to follow our [LMMA village program] rules.

(Interview with community conservation team member, Tanimbar Kei, March 2015).

Restrictive measures on where and what can be fished, and imposition of payments for seasonal fishing concessions to all outside fisher groups, undermines Mun residents’ particular relationship with outside Bugis fishers and so too their connection to those networks. Without active participation and opportunity for skills development for Mun residents, and without extra efforts to inform Mun residents of the importance of conservation measures for their livelihood security, they appear unlikely to compromise the existing safety nets they maintain through links to extensive Bugis diaspora networks.

## Reflecting on Strategic Leadership

### Aligning Adat Norms with Official Leadership Roles

Following community-wide recognition of the need to manage marine resources more sustainably, distinct new leadership roles and functions have formed out of encounters between community leaders and I-LMMA. The collaborations were used in different ways to promote not only collective interests but also the particular political interests of the *adat* group. The primary function of the administrative village government was locally understood to facilitate the flow of resources from state development projects to the village and to facilitate official engagements with external organizations, first and foremost with government authorities. On the other hand, internal village governance matters, especially social matters, still fell to the responsibility of the local *adat* leaders who used their norm-rooted authority to mold conservation and development programs to those customs and values.

With village leadership embedded in local dominant *adat* norms and with village government positions predominantly filled by *adat* core members, *adat* structures have been reinforced through I-LMMA’s intervention. Individuals from the *adat* group were unsurprisingly appointed to program coordinating positions following consultation with I-LMMA. The village secretary, for example, was a head of one of the 23 *rahan*, but alongside his official role in village government he also was the financial coordinator in the LMMA program. Similarly, the second program coordinator, who doubled as the focal area coordinator, is in line to inherit the position as head of his *rahan*, and also functioned as the village facilitator in the national community development program (PNPM). Only the third coordinator, being the Catholic leader, was not a kin-member of a *rahan*. However his position was also close to the *adat* core by marriage into one of the 23 *rahan* to which he had aligned himself over the years. All three of these individuals may not have had the same local authority as *adat* elders, however their alignment with *adat* means they enacted those norms in engaging with outside actors while drawing from technical skills that *adat* elders often lacked.

### Concessions of Adat Leadership Following I-LMMA Criteria

In collaborating with I-LMMA *adat* leaders made certain concessions regarding recruitment for the village LMMA program, which followed particular criteria that I-LMMA required to ensure broad participation. I-LMMA urged the inclusion of at least two women and a representative from Mun to be part of the core community conservation team. These criteria were met, however the selection of these individuals was strategic in that both women and the Mun representative who were eventually involved were each from *adat* lineages. I-LMMA’s recruitment criteria were also implicated in the selection of the three coordinators, by inclusion of a younger person among the three coordinating positions. This was in part to ensure sustainability of the program, to transcend generational divides and to involve individuals with the skills and learning potential of the younger generation. This led to the recruitment of the second coordinator, who later would also come to function as the focal area coordinator. Although it was likely that this person would come to play an important role in future *adat* leadership, being the first son of one of the *rahan* heads, his rapid rise into the coordinator position resulted from his selection by *adat* leaders following I-LMMA’s criteria. This initial exposure as a coordinator in the village LMMA program has led to similar coordinating roles being assigned to him in other unrelated projects, as for example the village facilitator under government sponsored rural development projects. As important as his *adat* association, was the fact that he exhibited exceptional skills in both communicating with authorities and mobilizing local resources and people to ‘get things done’ in village projects. The elevation of individuals to leadership positions appears then to be based on more than simply *adat* association, but also on personality, perceived skills and a proven track record. Although the initial opportunity to show these skills was through an I-LMMA intervention, *adat* leaders’ decision to build upon this has elevated his position into one of broader village planning and leadership.

To maintain collaboration with I-LMMA the *adat* leadership recognized the need to make important concessions in their recruitment of program leaders. Their adherence to I-LMMA’s recruitment criteria was somewhat consolidated by measures in the recruitment process that would prevent loss of *adat* representation. As a result in negotiating criteria and identifying individuals matching those, the candidates’ *adat* ties appeared to be of prominent, but not determinate, importance.

### Utilizing Outside Links to Strengthen Local Leadership Legitimacy

From its inception, the village LMMA program provided opportunities for prominent *adat* engagement. Beyond the primary gains of improved management of the island’s marine resources and the collective income generated from it, the collaboration over time proved beneficial to the position of *adat* leadership. First and foremost I-LMMA’s primary alignment with the *adat* social institutions meant resources and personnel were allocated largely through *adat* engagement. This strengthened the existing dominance of the *adat* core’s leadership in internal decision-making and patterns of participation within the village. It further reinforced the norms and values by which the community was internally governed. Recognizing that I-LMMA’s inception strategy involved a deliberate alignment with customary institutions, it is important also to note the relative weakness of the village government (*dinas*) structures in Tanimbar Kei, and indeed throughout Indonesia (Hidayat and Antlov 2004; Antlov and Eko 2012), and the fact that there was no other sub-group within Tanimbar Kei that would have the experience or governance legitimacy to accomplish much in the short term. Moreover, developing artificially established institutions is found in the literature to rarely take genuine root (Acheson 2006). In acknowledging these points, it may be suggested that I-LMMA had few other starting point options for collaboration beyond that with the established *adat* institutions. I-LMMA’s criteria for expanded representation appears then to have adapted an existing recruitment process dictated by *adat* interests to a more inclusive arrangement, rather than institutionalizing a (new) and arguably more democratic one that would in the short term undoubtedly have been less effective.

Secondly, the collaboration yielded official recognition of local *adat* among institutions beyond the community. Under district level legislation, subdistrict authorities accepted Tanimbar Kei’s ownership and management rights over the community’s marine territory based on *adat* claims to those areas. This provided for the first time a mechanism whereby Tanimbar Kei’s *adat* rules could be applied legitimately to outsiders, in, for example, its capacity under district law to sanction the intrusion of outside illegal fishers who were fishing without permission or using illegal fishing methods. The institutional channel through which these regulations were carried out was the administrative village government; however, the principles of local autonomy and ownership on which these regulations are founded reflected strong interests in the recognition of local *adat* norms. This was also evident in that all village government staff were also *adat* affiliated.

Within the village, the engagement with I-LMMA fortified local leadership and maintained legitimacy of *adat* institutions in times of rapid socio-economic and political change on the island, arising from dramatic changes through democratization and decentralization policies that recognized previously deprived customary community property rights and enhanced village autonomy. The close community engagement of I-LMMA’s grassroots approach, provided opportunity for the dominant *rahan* groups at the time to strengthen particular leadership constellations over other potential arrangements, in particular, the potential for more prominent leadership roles among minority migrant and Muslim household groups.

### Leadership Representation

Members from Mun households who were not related to the 23 *rahan* had little input in the decision-making processes. Although Mun households had a stronger seaward livelihood orientation, in part because as in-migrants they had no legitimate claim to land, their inclusion in decision-making about access to and use of marine areas remained low. Their participation was limited to consultation meetings that involved primarily informing Mun residents of plans to establish no-take zones for input around the implementation, rather than participation, at earlier planning and design stages. Mun residents’ reluctance to attend open meetings indicates that this exclusion was not apparently contested, and may have been chosen by Mun residents themselves. Their fishing and seaweed based livelihoods meant they could operate in spaces that did not impinge necessarily on others in the main village, and vice versa.^11^ The local divide between Mun and the main village, exacerbated by geographic separation, has over time resulted in a sense of autonomy amongst Mun residents, which they enacted in spaces that were made available to them by the dominant *adat* social group. As long as Mun residents’ livelihood activities were unaffected, there appeared little interest in representation in decision-making processes of the LMMA village program. This was to avoid compromising that autonomy they maintained on the periphery. I-LMMA’s measures for input across the village, through insistence that meetings be held in Mun or by encouraging invitations for Mun residents to participate in project activities, were thus not only subject to *adat* domination of village leadership, but also to the reluctance by Mun residents to become involved or speak up.

I-LMMA’s strategy was to gain community-wide interest through the dominant *adat* group. It is unsurprising then that interests were represented which primarily reflected their norms and ideals. The power differentials between groups within a village clearly play out in how decentralization and democratization of decision-making take shape (Béné *et al.*^2009^). As a consequence there appears very little direct engagement between peripheral groups and I-LMMA. The substantial marginalization of minorities leaves potentially important interest groups without meaningful roles.

In the context of marine conservation in Tanimbar Kei, given the strong seaward orientation of Mun residents, their lack of involvement in decision-making and implementation potentially compromises effective long-term management. Resistance from groups like Mun will likely emerge if future conservation measures were to significantly impinge on their livelihoods or social relationships. The LMMA village program is increasingly focusing on management of outside fishers, evident in the formulation of the village fishing regulations that stipulate sanctions for illegal or unlicensed fishing by outsiders. With a majority of such outside fishers being of Bugis origin, tensions between Mun and the main village are likely to develop. Gunawan and Visser (2012) similarly note the importance of such extensive diaspora networks for access of mobile fisher groups like the Bugis to particular fishing grounds, through interdependent patronage networks with ethnically related residents. They argue that such extensive socio-economic networks make borders, or place-based restrictive measures that are meant to control access, less effective, or what they term as ‘permeable’ (Gunawan and Visser 2012: 199). In Tanimbar Kei’s waters there is a recognized need to address the issue of illegal outside fishers. However the fact that the resource use regulations are enacted through village government, which is dominated by *adat* core representatives, and that the village LMMA program sees little close participation by Mun residents, implies that a potential means to engage more effectively with these outside fishers is eliminated.

## Conclusion

The article focused on the interactions between intra-village leadership constellations and external agencies. The case study sought to highlight the dynamics and the morphologies of local leadership, in customary leaders’ efforts to control implementation and benefit distribution so that projects are deemed useful locally. The study explicitly centers around these interactions to illustrate in particular the ability of leaders representing Tanimbar Kei’s *adat* core of 23 houses or *rahan* to dominate collaboration with an environmental NGO in a way that addressed concerns over environmental management while corroborating their local leadership position in the wider village.

The ongoing and effective collaboration with I-LMMA since 2005 functioned through direct links to the mediating *adat* core leadership. This provided the opportunity to address issues of environmental degradation that had significantly impacted all residents on the island. The program was channeled through the strong customary governance structure already in place. Evidently, the *adat* core and its leading families, were active in the recruitment of villagers and establishment of particular paths for defining and enhancing community resources and determining who could access them. The *adat* core group succeeded in developing means to enhance the external legitimation of their leadership positions, in order to manage marine resources within zones officially devolved to local governance (i.e., an area of four nautical miles). Despite this dominance there was significant evidence that the control over the LMMA village program was not entirely directed towards dominant group appropriation but also that needs of other groups were mediated and to a certain extent that wider benefit distribution occurred.

External organizations seeking to establish collaborative arrangements with communities must acknowledge that such partnerships come to function in arenas where on the one hand established interests are likely to divert new paradigms of practice, but on the other hand local leadership constellations have the potential to benefit from the introduction of more inclusive governance practices. Individuals in leadership positions, who mediate between community and NGOs such as I-LMMA, channel information and guide project implementation according to established social relationships and accountabilities in these communities. It is important not only to recognize how dominant leadership groups function in relation to heterogeneous community constituencies they represent, but also to identify particular constituencies that do not receive representation. Mun residents’ position, as primarily fishers, makes them an important stakeholder in discussions on marine resource management. However with the majority of Mun residents having limited kinship or other associations with the *adat* core group, their involvement remained highly peripheral despite some participation measures fostered through I-LMMA. These mechanisms for broad representation were defused partially by the influential *adat* leadership and partially by Mun residents’ own reluctance to face *adat* leadership. Without active contestation from Mun, their absence in the *adat* core dominated decision-making process is likely to remain unaddressed. It is plausible that resistance by Mun residents to the LMMA village program may arise if measures are introduced that increasingly restrict access to important marine resources for them or that compromise important social-economic and cultural networks they maintain with outside Bugis fishers. In identifying such emerging tensions and with sufficient adaptive capacity in the management framework, adjustments can be made to expand participation and prevent collapse.

The positive conservation outcomes in the LMMA village program’s first decade of operation, suggest that the domination of the *adat* core in Tanimbar Kei should not be interpreted to imply ‘failure’ or an unambiguous case of ‘elite capture’. This case supports arguments which suggest that cooperation by external agencies with dominant local groups and elite individuals provide valuable entry points for engaging communities in conservation and sustainable development initiatives. However, being aware of tensions and potential gaps in representation is critical to the adaptive local management and benefit distribution that would enable long-term sustainable management (Sutton and Rudd 2014). External mediation stands to play an important part in steering established governance patterns in more inclusive directions. Critical is the way that external agencies engage locally. The extent of knowledge, creativity, sensitivity and communicative ability of community-based outreach staff and their engagement with local culture brokers, will determine how effective negotiations across subtle socio-political divides occur, and how adaptive management structures will prove to be during shifts in such divides. In line with Cleaver’s (2012) argumentation for *bricolage* effects, the case study shows that a systematic focus on inclusion in locally based (and driven) interventions could bring about more effective resource governance.

## Footnotes

1 Law No. 22/1999 on regional autonomy and later through the revised Law No. 32/2004 on local government.

2 In the Indonesian context discussed in this article, local governance typically involves some combination of state-based administrative village (*desa dinas*) government and customary village (*desa adat*) norms, rules and values.

3 This is also reflected in the considerable body of literature devoted to addressing issues of elite capture in propoor natural resource management and development initiatives (Platteau 2004; Dasgupta and Beard 2007; Persha and Andersson 2014)

4 Henceforth. ‘I-LMMA’ will be used when referring to the NGO, while ‘LMMA’ will be used when referring to the village program.

5 In Maluku indigenous terms are officially used instead of the standard Indonesian government terminology noted here, namely *ohoi* for village and *ohoi soa* for hamlet. To allow for contextual comparison beyond Maluku cases, national terminology is used throughout this article.

6 This was evident from villagers’ response to significant and immediate losses in villagers’ income, food security and wellbeing following low seaweed yields in the recent past; access to their marine resources cushioned vulnerability rather than access to urban centers like Langgur.

7 These laws are woven in local myth and legend, and are believed to have been instilled by early Balinese migrants who sought to bring law and order to a disjointed system of tribal warfare (Adhuri 2006).

8 In the Kei islands three social groups exist, resulting from the historical sequence of immigration and rule on the islands. These include: ‘governors’ or ‘nobles’ believed to be descendants from the first immigrants from Bali and East Nusa Tenggara (*mel-mel*), indigenous land owners believed to be descendants of the autochthonous islands’ inhabitants (*ren-ren*), and the lowest social group believed to be descendants of prisoners or ‘slaves’ from early tribal battles (*iri-iri*).

9 I-LMMA’s four main focal areas include: The Kei Islands and Banda Islands in Maluku, and The Padaido Islands and Teluk Tanah Merah in Papua Province.

10 Arguably, recognition of Mun as a formal hamlet in the village administrative structure may have provided avenue for better representation of Mun interests.

11 Being located further from the main village, Mun residents used tidal zones for seaweed farming that by default were too far for villagers from the main village, meaning no tenure issues arose over seaweed farming.
